# Impact of SARS-CoV-2 Wuhan and Omicron Variant Proteins on Type I Interferon Response

**DOI:** 10.3390/v17040569

**Published:** 2025-04-15

**Authors:** Marija Janevska, Evelien Naessens, Bruno Verhasselt

**Affiliations:** 1 Department of Diagnostic Sciences, Ghent University, B9000 Ghent, Belgium; marija.janevska@ugent.be; 2 Department of Laboratory Medicine, Ghent University Hospital, B9000 Ghent, Belgium; evelien.naessens@uzgent.be

**Keywords:** SARS-CoV-2, interferon, luciferase, immune evasion, endothelial dysfunction, HUVEC, Betacoronavirus pandemicum

## Abstract

SARS-CoV-2 has demonstrated a remarkable capacity for immune evasion. While initial studies focused on the Wuhan variant and adaptive immunity, later emerging strains such as Omicron exhibit mutations that may alter their immune-modulatory properties. We performed a comprehensive review of immune evasion mechanisms associated with SARS-CoV-2 viral proteins to focus on the evolutionary dynamics of immune modulation. We systematically analyzed and compared the impact of all currently known Wuhan and Omicron SARS-CoV-2 proteins on type I interferon (IFN) responses using a dual-luciferase reporter assay carrying an interferon-inducible promoter. Results revealed that Nsp1, Nsp5, Nsp14, and ORF6 are potent type I IFN inhibitors conserved across Wuhan and Omicron strains. Notably, we identified strain-specific differences, with Nsp6 and Spike proteins exhibiting enhanced IFN suppression in Omicron, whereas the Envelope protein largely retained this function. To extend these findings, we investigated selected proteins in primary human endothelial cells and also observed strain-specific differences in immune response with higher type I IFN response in cells expressing the Wuhan strain variant, suggesting that Omicron’s adaptational mutations may contribute to a damped type I IFN response in the course of the pandemic’s trajectory.

## 1. Introduction

Severe Acute Respiratory Syndrome Coronavirus 2 (SARS-CoV-2) emerged in late 2019, triggering a global pandemic that profoundly impacted societies, economies, and healthcare systems. Compared to other RNA viruses, coronaviruses like SARS-CoV-2 have a notably larger genome (30 kb) [1] that not only encodes essential replication machinery but also a diverse set of accessory proteins that actively counteract host immune defenses (Table 1).

Early research prioritized adaptive immune responses [2,3,4], particularly antibody production and T cell-mediated immunity, due to the urgent need for a vaccine and therapeutic interventions. However, as our understanding of the virus evolved, increasing attention has been given to innate immune sensing pathways and their role in shaping disease outcomes. SARS-CoV-2’s ability to evade innate immunity facilitates viral replication and dissemination while contributing to severe clinical manifestations, including delayed immune responses, excessive inflammation, and endothelial dysfunction [5,6].

Despite the wealth of knowledge gained over the past few years, much of the research on SARS-CoV-2 immune evasion has been conducted using the original Wuhan strain and limited cellular models. There remains a critical gap in understanding how different SARS-CoV-2 strain proteins manipulate immune responses in endothelial cells specifically. Given the vascular complications associated with COVID-19, a detailed analysis of immune evasion mechanisms in endothelial cells is appropriate.

**Figure 1:**
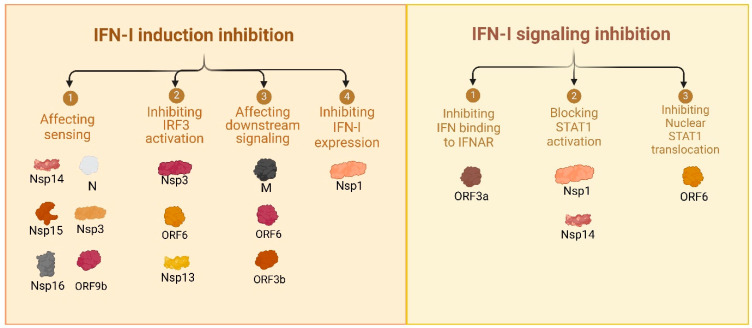
Affected steps of immune sensing by SARS-CoV-2 proteins (Wuhan strain) based on literature search (Table 1 and Table S1). SARS-CoV-2 proteins inhibit type I interferon (IFN-I) responses at multiple stages. (**Left panel**) IFN-I induction inhibition, including sensing (Nsp14, Nsp15, Nsp16, N, Nsp3), IRF3 activation (Nsp15), downstream signaling (ORF9b, M, ORF6, ORF3b), and IFN-I expression (Nsp1). (**Right panel**) IFN-I signaling inhibition, targeting IFNAR binding (ORF3a), STAT1 activation (Nsp1), and nuclear translocation of STAT1 (ORF6). These mechanisms highlight the multi-faceted immune evasion strategies of SARS-CoV-2. For references, see Table 1.

**Figure 2:**
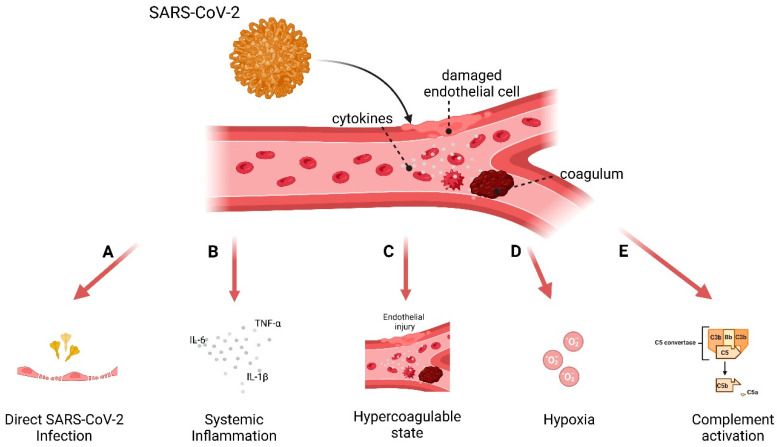
Mechanisms of endothelial dysfunction in COVID-19. The figure illustrates the key mechanisms driving endothelial dysfunction in COVID-19 and their downstream effects on the vascular system that can lead to fatal organ damage. (**A**) Direct SARS-CoV-2 infection: The viral spike protein binds to ACE2 receptors expressed on endothelial cells, facilitating viral entry and replication, leading to cellular damage [15,16,17]. (**B**) Systemic inflammation: Elevated pro-inflammatory cytokines, including IL-6, IL-1β, and TNF-α, activate endothelial cells, inducing an amplified inflammatory response [18,19,20,21]. (**C**) Hypercoagulable state: Endothelial injury promotes thrombin generation and platelet aggregation, resulting in the formation of thrombi and widespread vascular occlusion [12,22]. (**D**) Hypoxia: Reduced oxygen delivery due to severe respiratory distress exacerbates endothelial dysfunction, further impairing tissue oxygenation [23,24]. (**E**) Complement activation: Overactivation of the complement cascade causes endothelial damage and contributes to pro-thrombotic states through the generation of C3 and C5 convertases [17,25,26].

In this study, we conducted a comprehensive review to guide our global screening assay, assessing the impact of all known SARS-CoV-2 proteins from both the Wuhan and Omicron variant on IFN induction in cell lines. Next, we selected proteins from both variants to express in primary endothelial cells to assess their effect on IFN type I response. Pattern recognition receptors (PRRs) such as Toll-like receptors (TLRs) and retinoic acid-inducible gene I (RIG-I)-like receptors detect viral components like RNA species and activate downstream signaling cascades [7]. These pathways converge on mitochondrial antiviral-signaling protein (MAVS), TANK-binding kinase 1 (TBK1), and interferon regulatory factors (IRF3 and IRF7), leading to the production of type I interferons (IFN-I), such as IFN-α and IFN-β [8]. IFN-I responses initiate antiviral states in infected and neighboring cells. However, SARS-CoV-2 has evolved multiple strategies to evade IFN responses and sustain efficient replication (Figure 1). Approximately 0.5 to 1% of infected individuals succumb to COVID-19. Unlike other respiratory viruses, SARS-CoV-2 is distinguished by its severe vascular implications [5]. In COVID-19, endothelial dysfunction and vascular inflammation are associated with thrombo-inflammatory complications, such as increased vascular permeability, thrombosis, and systemic inflammation, often referred to as the “endothelitis” observed in severe cases [9,10,11], as well as a hypercoagulable state [12] that can lead to fatal organ damage (Figure 2). While SARS-CoV-2 primarily infects respiratory epithelial cells, it has become increasingly clear that endothelial cells play a crucial role in COVID-19 pathogenesis [13,14]. Endothelial cells (EC), which line the blood and lymphatic vessels, are critical regulators of vascular homeostasis. SARS-CoV-2 infects endothelial cells [15], initiating a cascade of events that impair EC function and exacerbate systemic inflammation.

## 2. Materials and Methods

### 2.1. Cells

HEK293T were cultivated in Iscove’s Modified Dulbecco’s Medium (IMDM) (Thermo Fisher Scientific, Merelbeke, Belgium) supplemented with 10% (*v*/*v*) heat-inactivated fetal calf serum (FCS, Hyclone, Thermo Fisher Scientific), 2 mM L-glutamine (Thermo Fisher Scientific), 100 U/mL penicillin, and 100 μg/mL streptomycin (Thermo Fisher Scientific).

Human umbilical cords were donated by mothers from the maternity ward after providing informed consent and approval by the Ghent University Hospital ethical committee (ONZ-2022-0272). HUVEC cells were freshly isolated as previously described [27]. They were cultured in Human Large Vessel Endothelial Cell Basal Medium (formerly Medium 200) (Thermo Fisher Scientific) supplemented with Large Vessel Endothelial Supplement (LVES) (Thermo Fisher Scientific), 2 mM L-glutamine (Thermo Fisher Scientific), and 100 U/mL penicillin and 100 μg/mL streptomycin (Thermo Fisher Scientific). Before cell plating, six-well plates were freshly coated with 5 μg/mL fibronectin solution (fibronectin powder, Sigma-Aldrich, Diegem, Belgium) by incubating them for 30 min at room temperature, followed by aspiration of the solution before cell plating. The isolated HUVECs were plated (3.5 × 10^5^ cells/well) and kept in culture on fibronectin-coated plates for a week and passaged twice at a seeding ratio 1:2, before being used for subsequent experiments.

### 2.2. Expression Constructs

Codon-optimized open reading frames and proteolytically mature nonstructural proteins (Nsps) of all 29 viral proteins from SARS-CoV-2 Wuhan and Omicron BA.1 strain were inserted into the pLVX-EF1alpha-IRES-Puro expression vector, with a Kozak sequence before each start codon and a 2X-Strep tag with a linker at either the N- or C-terminus. The only exception was ORF6, which was cloned from the Omicron BA.2 strain due to the presence of an additional D61L mutation unique to this variant. Additionally, Nsp3 from the Wuhan strain was not cloned due to technical limitations. These expression constructs were kindly provided by Dr Nevan Krogan [28,29].

In our current study, recombinant SARS-CoV-2 envelope proteins (S, M, and E) were expressed from expression plasmids rather than from subgenomic positive strand RNAs through the native viral infection pathway. As a result, their glycosylation, trafficking, and membrane localization may not fully recapitulate those observed during live viral infection, and this limitation should be considered when interpreting the functional effects of these proteins in this paper.

### 2.3. Dual-Luciferase Reporter Assay

The assay was optimized as described previously [30]. HEK293T cells seeded in 48-well plates (0.5 × 10^4^ cells/well) were co-transfected with 100 ng of reporter plasmids carrying an ISRE-promoter driving the Firefly luciferase activity (pISRE-F-luc), 20 ng of a pGAPDH-R-luc plasmid carrying GAPDH promoter driving Renilla luciferase activity as control of transfection, and 250 ng of a viral SARS-CoV-2 protein expressing plasmid. For the latter, strep II-tagged expression constructs encoding the 29 currently known SARS-CoV-2 proteins were used (Nsp1, Nsp2, Nsp3, Nsp4, Nsp5, Nsp6, Nsp7, Nsp8, Nsp9, Nsp10, Nsp11, Nsp12, Nsp13, Nsp14, Nsp15, Nsp16, S, ORF3a, ORF3c, E, M, ORF6, ORF7a, ORF7b, ORF8, ORF9b, N, ORF9c, and ORF10), i.e., variants from the original Wuhan strain and from Omicron BA.1 (kindly provided by Dr Nevan Krogan) strain were included. FuGENE HD (Promega, Leiden, The Netherlands) was used for all of the transfections per manufacturer’s instructions. Cells were treated with 1000 U/mL IFN-β or 500 U/mL IFN-α2 and harvested and lysed 8 or 24 h post-stimulation. Cell lysates were assayed for luciferase activity using the Dual-Glo luciferase assay system and GloMax^®^ Explorer microplate Luminometer (Promega). Firefly luciferase activity was normalized to cell viability and to Renilla activity to account for transfection efficiency. Results were expressed as remaining percentage activity compared to fully stimulated ISRE promoter, which was co-transfected with an empty control vector not carrying a viral protein. At least 3 biological replicates were included.

### 2.4. Cell Viability Assay

To measure metabolic activity affected by the individual SARS-CoV-2 viral proteins, transfected cells were lysed in passive lysis buffer at 8 h and 24 h post-transfection and analyzed using the CellTiter-Glo Luminescent Cell Viability Assay according to the manufacturer’s instructions and GloMax^®^ Explorer microplate Luminometer (Promega).

### 2.5. Production of Lentiviruses Encoding Single SARS-CoV-2 Viral Proteins

Lentiviruses were produced using the Strep II-tagged expression constructs encoding the ORF9b, Membrane, and Envelope proteins both from the Wuhan and the Omicron BA.1 strains. The lentiviral titer was measured by quantification of reverse transcriptase activity (RT) via real-time PCR [31]. HEK293T cells were also transduced and stained after permeabilization with primary anti-Strep antibody (NWSHPQFEK Tag Antibody, mAb, Mouse, GenScript, Boechout, Belgium) and secondary anti-mouse APC antibody (A-865, Thermo Fisher Scientific) as controls.

### 2.6. Transduction of HUVEC and IFN Stimulation

A total of 250,000 cells per well were seeded in fibronectin-coated 6-well plates. A day was allowed for cell attachment before transduction. Cells were transduced with lentiviruses expressing the selected SARS-CoV-2 proteins, either from the Wuhan or the Omicron variant, in the presence of 1 μg/mL polybrene to aid the transduction (Figure S1). The cells were stimulated with 500 IU/mL IFN-α2 or 1000 UI/mL IFN-β 24 h post-transduction. Non-stimulated transduced cells were kept for each condition, and non-transduced cells were also included as controls. At 24 h after the stimulation, the cells were lysed using a Qiazol lysis buffer and kept at −80 °C overnight to aid the lysis. mRNA was extracted from cell lysates the next day using the QIAGEN RNeasy Mini kit (QIAGEN, Hilden, Germany), the mRNA samples were measured, and qPCR was performed.

### 2.7. RT-qPCR

In brief, mRNA was extracted from HUVEC cells using the QIAGEN miRNeasy Mini kit according to the manufacturer’s instructions. RNA (max 1 μg) was subsequently treated with amplification-grade DNAse I (Life Technologies, Merelbeke, Belgium) and used for synthesis of cDNA with Superscript III reverse transcriptase and random primers (Life Technologies). Depending on the gene to be measured, cDNA was subsequently diluted 3× (for target genes: IFNB1, IFNA2, and IFIT1) and 15× (for reference genes: ACTIN, RPL13A, YWHAZ, and UBC) with Nuclease-free water (Ambion, Life Technologies). A total 5 μL of the diluted cDNA was then used for qPCR. For qPCR, LightCycler 480 SYBR Green I Master mix (Roche Diagnostics, Vilvoorde, Belgium) was used in final reaction of 15 μL. qPCR reactions were performed in 384-well plates (LightCycler 480 Multiwell Plates 384, white, Roche Diagnostics) on the Light Cycler 480 II instrument (Roche Diagnostics).

All samples were measured in duplicate. A non-template control (nuclease-free water instead of cDNA) and a serial 10-fold dilution of standard curve was used. The cDNA for the standard curve was synthesized using mRNA from poly(I:C) stimulated PBMCs, and this standard curve was included for the measurement of each gene on the plate. Melting curve analysis for IFIT1, IFNA2, and IFNB1 was performed and showed a single peak. Calibrated normalized relative quantities (CNRQs) were calculated for each target gene in each sample based on the obtained Cq values, with the qBase Software 2.0 (Biogazelle, CellCarta, Montreal, QC, Canada), using YWHAZ, ACTIN, RPL13A, and UBC as reference genes and using target- and run-specific amplification efficiencies.

Primers used for qPCR are as follows: UBC Fwd (sense) 5′-ATTTGGGTCGCGGTTCTTG-3′, UBC Rev (antisense) 5′-TGCCTTGACATTCTCGATGGT-3′, YWHAZ Fwd (sense) 5′-CTTTTGGTACATTGTGGCTTC AA-3′, YWHAZ Rev (antisense) 5′-CCGCCAGGACAAACCAGTAT-3′, ACTIN Fwd (sense) 5′-TGACCCAGATCATGTTTGAGA-3′, ACTIN Rev (antisense) 5′-AGAGGCGTACAGGGATAGCA-3′, RPL13A Fwd (sense) 5′-CCTGGAGAAGAGGAAAGAGA-3′, RPL13A Rev (antisense) 5′- TTGAGGACCTCTGTGTATTTGTCAA-3′, IFIT1 Fwd (sense) 5′-GATCTCAGAGGAGCCTGGCTAA-3′, IFIT1 Rev (antisense) 5′-TGATCATCACCATTTGTACTCATGG-3′, IFNA2 Fwd (sense) 5′-GTGAGGAAATACTTCCAAAGAATCAC-3′, IFNA2 Rev (antisense) 5′-TCTCATGATTTCTGCTCTGACAA-3′, IFNB1 Fwd (sense) 5′-GCTTCTCCACTACAGCTCTTTC-3′, IFNB1 Rev (antisense) 5′-CAGTATTCAAGCCTCCCATTCA-3′.

All primers were purchased from IDT (Integrated DNA Technologies, Europe Branch, Leuven, Belgium).

### 2.8. Figure Generation

**Figure 3:**
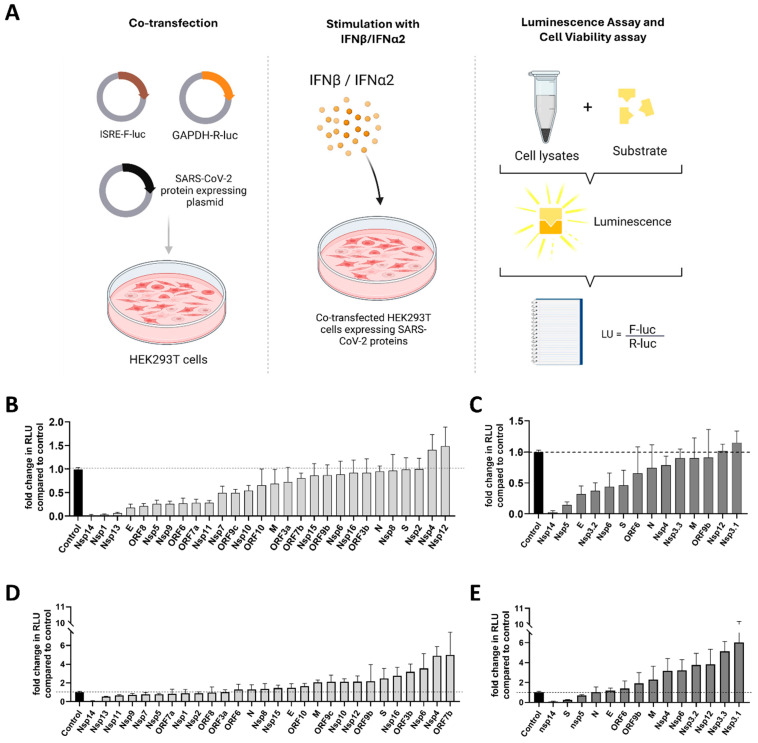
Functional screening of SARS-CoV-2 proteins from Wuhan and Omicron BA.1 variants on their ability to suppress or induce immune response. (**A**) Schematic representation of the experimental workflow. HEK293T cells were co-transfected with Firefly luciferase reporter plasmid under the control of an ISRE promoter (pISRE-F-luc), a Renilla luciferase plasmid (pGAPDH-R-luc) as a control for transfection efficiency, SARS-CoV-2 protein expression plasmids encoding individual SARS-CoV-2 proteins from the Wuhan (**B**,**D**) or Omicron BA.1 (**C**,**E**) variants, and stimulated with IFN-β or IFN-α2. Firefly luciferase activity was measured using a Dual-Luciferase Reporter Assay and normalized to Renilla activity and cell viability. Data represent the mean ± SEM from three independent experiments, each including two technical replicates. Cells were stimulated with IFN-β (**B**,**C**) or IFN-α2 (**D**,**E**). Results are presented as fold-change in relative luminescence units (RLU) compared to fully stimulated ISRE promoter activity in cells co-transfected with an empty control vector (dotted lines). Panels (**B**,**C**): IFN-β-stimulated ISRE promoter activity. Both Wuhan and Omicron BA.1 proteins show a range of inhibitory effects, with certain proteins (e.g., Nsp1, Nsp14) inducing significant suppression of promoter activity. Panels (**D**,**E**): IFN-α2-stimulated ISRE promoter activity. Similar patterns of suppression are observed for both variants, with Nsp1 and ORF9b showing the strongest inhibition of ISRE-driven luciferase expression.

Figure 1, Figure 2, Figure 3A and Figure S1 were created using the BioRender, version 04 (Toronto, ON, Canada) application after obtaining the appropriate license for publication. All graphs were generated using GraphPad Prism 8.0 software (Boston, MA, USA).

## 3. Results

### 3.1. Comprehensive Review of SARS-CoV-2 Proteins and Their Immune-Modulatory Effects

One of the defining features of SARS-CoV-2 pathogenesis is its ability to suppress and delay IFN responses.

To provide a comprehensive understanding of the immune-modulatory effects of SARS-CoV-2 proteins, we systematically reviewed the available literature to create a detailed reference (Table 1). This table catalogs the functions of all 29 currently known viral proteins on host pathways, highlighting their diverse mechanisms of action, including immune evasion, host machinery manipulation, and inflammatory response modulation. From this, we reanalyzed and organized the data into a second framework (Table 2), where viral proteins were categorized based on specific immune evasion strategies. These strategies include hijacking host protein synthesis machinery, protecting viral RNA, protecting viral proteins, facilitating immune suppression, and subverting innate immune pathways. This integrative analysis offers detailed insights into the multitiered immune evasion tactics employed by SARS-CoV-2 and sets a foundation for further exploring these viral mechanisms.

**Table 1:** SARS-CoV-2 Wuhan strain viral protein’s effect on host.

Protein	Effect on Host
Spike (S)	▫ masks its epitope by glycosylation and forming a “sugar shield” [32,33,34,35]. ▫ activates NF-κB via ACE2 and increases IL-6, MCP-1, ICAM-1, PAI-1, P-selectin, and complement [17,36,37]. ▫ increases ROS, suppresses PI3K/AKT/mTOR and induces autophagy, apoptosis, and cytokine release [38]. ▫ binds sialylated glycans, causing hemagglutination and microvascular occlusion [39].
Nucleocapsid (N)	▫ binds TRIM25, blocking RIG-I ubiquitination and activation [40,41,42]. ▫ inhibits IRF3 phosphorylation and its nuclear translocation [41,43,44]. ▫ enhances MAVS SUMOylation and reduces downstream phosphorylation of TBK1, IKKα, and IRF3 [43,45]. ▫ inhibits NF-κB activation by disrupting the assembly of the TAK1-TAB2/3 complex [46]. ▫ localizes to mitochondria, upregulates Complex I/III proteins, and increases mitochondrial ROS [19]. ▫ inhibits stress granule formation by sequestering G3BP1/2 [42,47].
Membrane (M)	▫ interacts with the MAVS and impairs recruitment of TRAF3, TBK1, and IRF3 [48]. ▫ exhibits strong IgM and IgG immunoreactivity, making it highly antigenic and highly inflammatory [49]. ▫ interacts with ATPase subunits reducing their cellular activity, which increases apoptosis [50].
Envelope (E)	▫ interacts with PALS1, disrupts epithelial barriers, causes inflammation and tissue remodeling [51]. ▫ forms viroporins, which disrupt ion gradients and membrane potential, and triggers pyroptosis-like cell death, upregulating TNF-α and IL-6 [52]. ▫ binds TLR2; activates NF-κB/ERK; and induces IL-6, TNF-α, CCL3, and CXCL8 [53,54,55]. ▫ downregulates CD1d, disrupts post-ER maturation and trafficking, and prevents iNKT activation [56]. ▫ activates NLRP3 inflammasome and amplifies inflammatory cytokines [55]. ▫ interacts with the epigenetic regulators BRD2 and BRD4 and alters host gene transcription [57].
Nsp1	▫ inhibits translation and promotes degradation of host mRNA, while sparing non-coding RNAs and viral transcripts [58,59]. ▫ causes nuclear retention of host mRNAs by interacting with NXF1-NXT1 [60,61]. ▫ blocks STAT1 phosphorylation and suppressing downstream signaling [62,63,64]. ▫ downregulates NKG2D ligands (MICA, ULBP1, ULBP2) on infected cells, reducing NK cell recognition [65]. ▫ displaces RCAN3 from calcineurin A to activate the NFAT pathway and upregulates DDX5 [64].
Nsp2	▫ binds to the GIGYF2-4EHP complex and blocks translation of IFN-β [66,67]. ▫ activates the NF-κB pathway by phosphorylating the p65 subunit [68].
Nsp3	▫ interacts with REEP5 and TRAM1 to facilitate double-membrane vesicle (DMV) formation shielding replication–transcription complex (RTC) [69]. ▫ the PLpro domain deubiquitinates, deISGylates, and deSUMOylates host proteins, disrupting RIG-I, STING, and NF-κB pathways [70,71,72]. ▫ binds FMRPs, displacing UBAP2L and blocking stress granule formation [73]. ▫ the SUD domain binds host DNA/RNA G4s, disrupting mRNA translation and splicing [69,74].
Nsp4	▫ interacts with BCL2 proteins, causing mitochondrial damage and mtDNA release, and activating cGAS-STING and NLRP3 inflammasomes [75]. ▫ forms replicopores with Nsp3 for viral RNA transport while shielding it from host immune sensors [76].
Nsp5	▫ promotes SUMOylation of MAVS and activates NF-κB, increasing IL-1β, IL-6, and TNF-α production [77]. ▫ cleaves RIG-I and MAVS, disrupts G3BP1, and prevents TBK1/IRF3 phosphorylation [78,79,80]. ▫ cleaves NEMO, TRMT1, and MAGED2, disrupting IFN production [81,82,83]. ▫ recruits HDAC2 via IRF3, deacetylates CIITA and downregulates MHC II expression [57,84,85]. ▫ disrupts Bcl-2 expression by binding to G4 structures in its promoter and upregulates apoptosis [86].
Nsp6	▫ restricts autophagosome expansion by inhibiting lysosomal fusion and acidification via SIGMAR1 [87,88,89]. ▫ activates NF-κB via TAK1, NEMO, and TRIM13, increasing cytokine production [90]. ▫ suppresses JAK-STAT and MAVS pathways by inhibiting IRF3 and STAT1/STAT2 phosphorylation [44,91]. ▫ activates NLRP3 inflammasome and causes cleavage of gasdermin D and IL-1β/IL-18 [92]. ▫ causes mitochondrial damage via ATP reduction, impairing calcium handling and inducing ROS production [93].
Nsp7	▫ disrupts RIG-I/MDA5-MAVS, TLR3-TRIF, and cGAS-STING pathways, by preventing complex formation and reducing IRF3 phosphorylation [94]. ▫ binds to selenoprotein S, disrupting cytokine suppression and ER-associated degradation [95]. ▫ binds HLA-DRB1, activates Tregs, and suppresses CD4+/CD8+ memory responses [96]. ▫ causes mitochondrial damage by increasing ROS and decreasing ATP [97].
Nsp8	▫ inhibits MAVS signaling by interacting with MDA5 and blocking IRF3 and TBK1 [98,99]. ▫ causes cardiomyocyte damage due to disruption in ATP production, apoptosis, and calcium handling [50]. ▫ damages mitochondria by reducing membrane potential and increasing ROS production [100].
Nsp9	▫ suppresses NF-κB signaling by impairing p65 nuclear transport via NUP62, downregulating TBK1 activity, promoting TRIM27-mediated TBK1 degradation, and reducing RIG-I expression [101,102,103].
Nsp10	▫ part of the capping machinery, preventing viral RNA recognition by host immune sensors [104,105].
Nsp11	▫ unknown effects.
Nsp12	▫ suppresses alternative splicing of host ISGs by interacts with splicing factors SLU7, PPIL3, and AKAP8 [106]. ▫ prevents IRF3 nuclear translocation without impairing its phosphorylation [107].
Nsp13	▫ stops antiviral IFN signaling and inflammation by blocking IRF3, NF-κB, and STAT1/STAT2 activation [63,108,109].
Nsp14	▫ blocks nuclear translocation of IRF3 [110]. ▫ activates canonical NF-κB signaling via IMPDH2 interaction [111,112,113]. ▫ evades Viperin recognition by removing the ddhCMP from viral RNA [114]. ▫ inhibits the TCA cycle of the host by interacting with sirtuin5 (SIRT5) and affecting the energy supply system [115].
Nsp15	▫ prevents IRF3 phosphorylation by binding to TBK1 and to karyopherin alpha 1 (KPNA1) [110,116]. ▫ degrades dsRNA intermediates to prevent recognition by dsRNA sensors [117,118].
Nsp16	▫ methylates the ribose of viral mRNA to mimic host mRNA, evading detection by IFIT proteins and RIG-I-like receptors (e.g., MDA5 and RIG-I) [119,120].
ORF3a	▫ inhibits STAT1 phosphorylation [44]. ▫ activates NLRP3 inflammasome via NF-κB activation [121,122]. ▫ downregulates MHC-I expression by inhibiting global protein trafficking to the cell surface [123]. ▫ activates both extrinsic and intrinsic apoptotic pathways [124]. ▫ increases viral particle release by rerouting tetherin to late endosomes/lysosomes [125]. ▫ inhibits autophagy and promotes lysosomal exocytosis and viral egress via ion channels [126,127,128,129].
ORF6	▫ binds importin KPNA2, blocking nuclear translocation of IRF3 and ISGF3 (STAT1/STAT2/IRF9 complex) [44,130]. ▫ binds with Rae1 in the cytoplasm [105] and binds the Nup98-RAE1 complex in the nucleus [131,132,133,134], thus blocking IRF3 and STAT1 nuclear translocation. ▫ directly binds STAT1, preventing its nuclear localization [135]. ▫ targets TRIM25 for proteasomal degradation, inhibiting RIG-I activation [136]. ▫ upregulates IL11 and WNT5A, activating STAT3 signaling and promoting fibrotic inflammatory responses [137].
ORF7a	▫ blocks STAT2 phosphorylation [138]. ▫ induces ER stress and apoptosis by recruiting BclXL to the ER, activating PERK-eIF2α-CHOP pathway [139]. ▫ activates the NF-κB pathway by interacting with TAK1 and NEMO [90]. ▫ downregulates MHC-I expression by delaying its export from ER [123]. ▫ induces autophagy but blocks autophagic flux [140]. ▫ prevents the incorporation of SERINC5 into virions [141].
ORF7b	▫ promotes TNF-α-induced apoptosis via activation of caspase-8 [142]. ▫ downregulates IFIT1 and TRIM22 while upregulating proinflammatory cytokines [143]. ▫ binds to MAVS and suppresses RIG-I pathway [144]
ORF8	▫ downregulates MHC-I by targeting them for degradation [145]. ▫ reduces antibody-dependent cytotoxicity (ADCC) by binding to CD16a on monocytes and NK cells [146]. ▫ upregulates proinflammatory cytokines IL-6, CCL3, CCL5, and CXCL10 [147]. ▫ attenuates complement activation by binding to C3 and C3b [148]. ▫ acts as a histone mimic to downregulate ISGs expression [149].
ORF9b	▫ targets TOM70 at mitochondria, suppressing TBK1 phosphorylation [150]. ▫ blocks NEMO ubiquitination (a NF-κB essential modulator), interfering with the RIG-I/MAVS pathway [151]. ▫ directly interacts with RIG-I, MDA5, MAVS, TRIF, STING, and TBK1, impeding the IRF3 phosphorylation [152]. ▫ inhibits MCL1 and induces mitochondrial DNA (mtDNA) release [75]. ▫ impairs cardiomyocytes metabolism by reducing ATP production and enhancing glycolysis [153]. ▫ blocks intracellular trafficking of immune receptors by blocking MARK2 activity via the KA1 domain [154]. ▫ upregulates fibrinogen α, β, and γ genes (pro-thrombotic factors) while downregulating albumin [155].
ORF10	▫ interacts with NIX and LC3B inducing mitophagy and degrading MAVS [156]. ▫ binds STING, which blocks cGAS-STING pathway and autophagy [157].

**Table 2:** Immune evasion strategies by SARS-CoV-2 Wuhan strain.

Strategy	Mechanism of Action
Hijacking protein synthesis machinery	Rapid shutdown of host mRNA translation: Nsp1 shuts down host protein mRNA translation [58]. Nsp2 binds GIGYF2-4EHP complex and suppresses host translation [67,158]. Nsp3—the SUD domain binds host DNA/RNA G4s, disrupting mRNA translation, stability, and splicing [74]. Nsp12 suppresses alternative splicing of host immune-related genes by interacting with splicing factors SLU7, PPIL3, and AKAP8 [106]. Envelope (E) alters host gene transcription by interacting with epigenetic regulators BRD2 and BRD4 [57]. ORF8 acts as a histone mimic to downregulate ISGs expression [149]. Increased production of key protein: ORF9b, ORF6, and Nucleocapsid (N) proteins are overexpressed in the first hours of infection [159].
Protecting viral mRNA	CpG deficiency: SARS-CoV-2 has the most severe CpG deficiency among betacoronaviruses, evading degradation by zinc finger antiviral protein (ZAP) [160]. Modifying viral RNA: Viral mRNA is capped using Nsp10 [105], methylated by Nsp16 [120], and Nsp14 [114] removes ddhCMP from viral RNA to avoid recognition, mimicking host mRNA to escape recognition by RIG-I, MDA5, IFITs, and Viperin. RNA cleavage: Nsp15 selectively cleaves viral RNA at poly-U sequences to avoid detection by host sensors and prevents dsRNA accumulation [117,118].
Protecting viral proteins	Formation of double-membrane vesicles (DMVs) to protect replication–transcriptase complex (RTC): Nsp3 [76], Nsp4 [76], and Nsp6 [87,89] are involved in DMVs formation, ensuring the RTC is protected (Nsp7 and Nsp8 act as co-factors for Nsp12 (RdRp), Nsp13 (helicase), Nsp14 (exonuclease), Nsp15 (endonuclease), and Nsp16 (2′-O-methyltransferase)), connecting to the ERGIC and Golgi apparatus during maturation [161]. Masking viral proteins with glycans: The Spike protein is heavily glycosylated, masking immunogenic viral epitopes [33,34,35].
Safe Release of virions	Tetherin Inhibition: ORF3a increases viral particle release by rerouting tetherin to late endosomes/lysosomes [125]. ORF7a inhibits BST-2 (tetherin) activity, aiding in the release of mature virions [162]. Lysosomal Exocytosis and Viral Egress: ORF3a inhibits autophagy and promotes lysosomal exocytosis, as well as forming ion channels mediating viral egress [126,127,129]. SERINC5 antagonism: ORF7a antagonizes SERINC5 and prevents its incorporation into virions [141].
Immune modulation	Inhibiting antigen presentation: MHC-I: ORF3a [123], ORF8 [145], and ORF7a [123] downregulate MHC-I expression. MHC-II: Nsp5 recruits HDAC2, which deacetylates CIITA and downregulates MHC-II, impairing CD4+ T cell activation [84,85]. RIG-I/MAVS pathway inhibition: Nucleocapsid (N) binds TRIM25, blocking RIG-I activation [40,41,42]. Membrane (M) interacts with MAVS, directly impairing TRAF3, TBK1, and IRF3 recruitment [48]. Nsp3—the PLpro domain deubiquitinates RIG-I [70,72]. Nsp5 degrades MAVS and RIG-I and prevents TBK1/IRF3 phosphorylation [78,79], while Nsp9 targets TBK1 for degradation [101,102]. Nsp8 interacts with MDA5 and blocks IRF3 and TBK1 [98,99]. Nsp12, Nsp14, and Nsp15 inhibit IRF3 nuclear translocation [107,110,116]. ORF9b interacts with TOM70 and prevents MAVS activation [152,159]. Nsp6 [163] and Nsp13 [63] block IRF3 nuclear translocation by disrupting the IKKε-TBK1 complex. ORF6 inhibits IRF3 nuclear translocation by sequestering Rae1 [164], blocking the Nup98-Rae1 complex [132] and binding importin KPNA2 [130]. It also targets TRIM25 for degradation, inhibiting RIG-I activation [136]. ORF7b binds to MAVS and suppresses RIG-I pathway [144]. ORF10 degrades MAVS by interacting with NIX and LC3B and inducing mitophagy [156]. JAK-STAT pathway inhibition: Nsp1 [63] and ORF3a [44] inhibit STAT1 phosphorylation. ORF6 binds importin KPNA2, blocking nuclear translocation of ISGF3 [130], and binds STAT1 directly [135]. ORF7a blocks STAT2 phosphorylation [138]. Nsp6 blocks STAT1/STAT2 phosphorylation [163]. TLR pathway: Nsp 7 disrupts TLR3-TRIF complex formation [94]. Nsp9 suppresses TLR activation and subsequent NF-κB signaling by impairing p65 nuclear transport via NUP62 [101]. Envelope (E) binds to TLR2 and activates NF-κB [53]. Proinflammatory Cytokine Induction: NF-κB activation: Spike (S) via ACE2 [17,36,37]; Nsp2 via p65 phosphorylation [68]; Nsp6 and ORF7a via TAK1, NEMO, and TRIM13 [90]; Nsp14 via IMPDH2 interaction [111]; Envelope (E) via TLR2 [53]; and Nsp5 via MAVS SUMOylation and NEMO cleavage [77]. Other: Nucleocapsid (N) upregulates Complexes I/III and increases ROS production [165]; inhibits stress granule formation leading to robust cytokine production [42,47]. ORF8 upregulates proinflammatory cytokines IL-6, CCL3, CCL5, and CXCL10 [166]. Inflammasome activation: Spike [167], Nsp6 [93], nsp7 [97], and nsp8 [100] activate inflammasome via increased ROS production. Envelope (E) and Nsp4 activate the NLRP3 inflammasome via mitochondrial damage [75]. Nsp6 activates the NLRP3 inflammasome via caspase-1 [92]. ORF3a activates NLRP3 inflammasome via NF-κB activation [121]. NK and T cell function modulation: Nsp1 downregulates NKG2D ligands (MICA, ULBP1, ULBP2) on infected cells, reducing NK cell recognition [65]. Nsp7 binds HLA-DRB1, activates Tregs, and suppresses CD4+/CD8+ memory responses [96]; it also binds to selenoprotein S and inhibits cytokine suppression [95]. ORF8 reduces antibody-dependent cytotoxicity (ADCC) by binding to CD16a on monocytes and NK cells [146].

For example, within the first hour of infection, Nsp1, Nsp2, and Envelope (E) act together to suppress host mRNA translation but through distinct pathways. Nsp1 achieves this by globally shutting down host protein synthesis, facilitating rapid viral replication; Nsp2 suppresses translation by binding the GIGYF2-4EHP complex; while the Envelope (E) protein manipulates host transcription through interactions with epigenetic regulators, redirecting cellular machinery to favor viral protein production. To protect its replication machinery, it employs Nsp3, Nsp4, and Nsp6 to form the replication–transcription complex (RTC), while many other viral proteins suppress immune signaling and antigen presentation in a coordinated manner. A plethora of proteins block the RIG-I/MAVS pathway through deubiquitinating or degrading a crucial signaling molecule or directly binding to a protein like IRF3 and preventing its nuclear translocation. SARS-CoV-2 assures its virion release by directly antagonizing tetherin via ORF7a or rerouting tetherin to endosomes and lysosomes via ORF3a.

Beyond IFN signaling, SARS-CoV-2 modulates antigen presentation to avoid immune detection. Proteins such as ORF3a, ORF7a, and Nsp5 independently suppress MHC-I and MHC-II expression. Furthermore, SARS-CoV-2 modulates natural killer (NK) cell and T cell responses. Nsp1 downregulates NKG2D ligands, reducing NK cell recognition of infected cells, while ORF8 binds CD16a on monocytes and NK cells, impairing antibody-dependent cytotoxicity (ADCC). Nsp7 binds HLA-DRB1 and activates regulatory T cells, suppressing CD4+ and CD8+ memory responses and cloaking infected cells from adaptive immune responses. These strategies impair cytotoxic T-cell recognition of infected cells, contributing to prolonged viral persistence.

### 3.2. Functional Screening of SARS-CoV-2 Wuhan and Omicron Strain Proteins for Impact on Innate Immune Sensing

Despite significant progress in understanding SARS-CoV-2 immune evasion strategies, most research has been focused on the original Wuhan strain (Table 1 and Table 2). Mutations in the Spike (S) protein, but also in accessory and non-structural proteins, have affected the virus strain phenotype tremendously [35]. Therefore, a re-evaluation of variant protein effect on viral infectivity, immune evasion, and pathogenicity is warranted.

In this study, we performed a global screening of the SARS-CoV-2 viral proteins from both the Wuhan and Omicron strains to assess their impact on type I interferon response. In HEK293T cells, we used a dual-luciferase reporter assay and Strep II-tagged expression constructs, coding for the 29 currently known SARS-CoV-2 proteins (Nsp1, Nsp2, Nsp3, Nsp4, Nsp5, Nsp6, Nsp7, Nsp8, Nsp9, Nsp10, Nsp11, Nsp12, Nsp13, Nsp14, Nsp15, Nsp16, S, ORF3a, ORF3c, E, M, ORF6, ORF7a, ORF7b, ORF8, ORF9b, N, ORF9c, and ORF10) from the original Wuhan strain and from the Omicron BA.1. strain (Figure 3A).

Several proteins are highly conserved and have no distinct variants between the two strains. Examples are the Nsp7-Nsp8 complex, the helicase (Nsp13), the endoribonuclease (Nsp15), and the 2′-O-methyltransferase (Nsp16), which are all part of the RTC. The impact of all viral proteins on a major branch of innate immunity, ISRE (IFN/pro-inflammatory cytokine induction by RIG-I-like receptors (RLRs)), was analyzed by measuring luciferase activity driven by stimulation of the ISRE promoter in quantitative reporter assays. Stimulation by type I IFNs (IFN-α2 and IFN-β) was titrated, quantified, and optimized beforehand using quantitative firefly luciferase reporters controlled by the respective promoters.

Stimulation with IFN-α2 and IFN-β revealed that activation of the ISRE is strongly repressed by Nsp1, Nsp5, Nsp14, and ORF6 across both strains (Figure 3B–E), underscoring their conserved immune evasion functions. Additionally, proteins such as Nsp9, Nsp11, Nsp13, ORF7a, and ORF8 from the Wuhan strain (identical protein in Omicron) displayed strong IFN inhibition. Intriguingly, when stimulated by IFN-β (Figure 3B,C) the Omicron Spike protein, carrying 32 additional mutations, exhibited enhanced suppression of IFN responses, irrespective of the stimulus of either IFN-β (Figure 3B,C) or IFN-α2 (Figure 3D,E).

**Figure 4:**
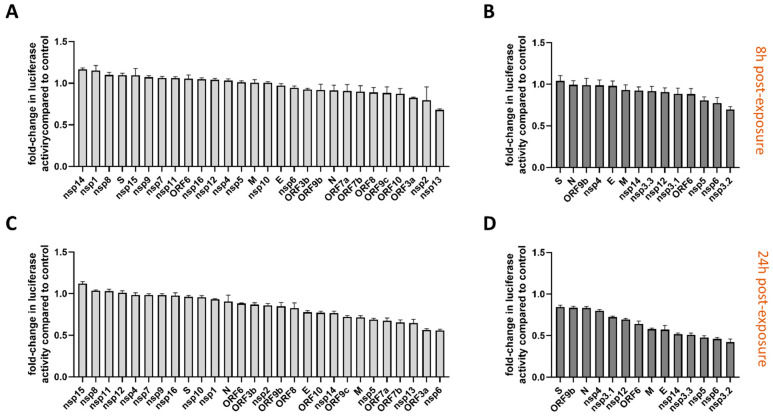
Cytotoxicity of individual SARS-CoV-2 proteins from Wuhan and Omicron BA.1 variants. HEK293T cells were transfected with expression vectors encoding individual SARS-CoV-2 proteins from the Wuhan (**A**,**C**) or Omicron BA.1 (**B**,**D**) variants. Cell viability was measured at 8 h (**A**,**B**) and 24 h (**C**,**D**) post-transfection using the CellTiter-Glo^®^ Luminescent Cell Viability Assay. The luminescent signal reflects ATP levels, proportional to viable cells (Supplementary Materials). Fold-change in luminescence activity (absolute luminescent units (ALU), see Figure S1) was calculated compared to empty vector controls (n = 2 for each experiment), included to account for transfection effects. Panels (**A**,**C**) show cytotoxicity profiles of individual Wuhan variant proteins. Minimal reductions in cell viability were observed at 8 h post-transfection (**A**), while some proteins exhibited mild to moderate toxicity by 24 h (**C**). Panels (**B**,**D**) show cytotoxicity profiles of individual Omicron BA.1 variant proteins. Similar to the Wuhan variant, most proteins showed minimal toxicity at 8 h (**B**). At 24 h (**D**), a subset of proteins induced moderate reductions in cell viability compared to the empty vector control. Data represent the mean ± SEM of fold-change in luminescence activity compared to control from three independent experiments, each with two technical replicates.

Additionally, we screened for cell viability at 8 and 24 h post-transfection to evaluate the toxic effect of each viral protein on the viability of the cells (Figure 4). At 8 h post-transfection, both Wuhan (Figure 4A) and Omicron BA.1 (Figure 4B) variants demonstrated minimal cytotoxic effects, as indicated by the near-complete preservation of ATP levels in transfected cells. This suggests that the early expression of individual viral proteins does not substantially impact cell viability under these conditions. However, by 24 h post-transfection (Figure 4C,D), time-dependent cytotoxic effects became apparent. Certain proteins, like Nsp3, Nsp6, and ORF3a, exhibited mild to moderate reductions in cell viability, indicative of their potential to disrupt cellular processes over time.

It is important to note that the variability in expression levels, post-translational modifications, and metabolic conditions can influence the cytotoxicity observed in cell lines. The goal of this screening was to identify proteins with substantial cytotoxicity that could confound downstream analyses, not to quantify and rank the cytotoxicity of individual proteins.

### 3.3. Model to Study Vascular Impact: Immune Response in Endothelial Cells

In SARS-CoV-2’s pathology, endothelial dysfunction and coagulation disorders occur, leading to sometimes fatal outcomes. Since ACE2 receptors are also expressed in endothelial cells (ECs), infection by SARS-CoV-2 occurs. Therefore, we investigated the effect of selected viral proteins on the expression of type I interferon pathways in human ECs from umbilical vein (HUVECs), expressing viral proteins by lentiviral transduction.

From both the Wuhan and the Omicron BA.1 strains, we selected the Envelope, the Membrane, and the ORF9b proteins to further compare their effect on human primary endothelial cells, because of their features (see Table 1) and a possible connection to cardiovascular complications and coagulopathies [75,153,155,168]. We could not test the ORF6 protein since expression of ORF6 from both variants was toxic and severely affected the viability of the primary endothelial cells.

**Figure 5:**
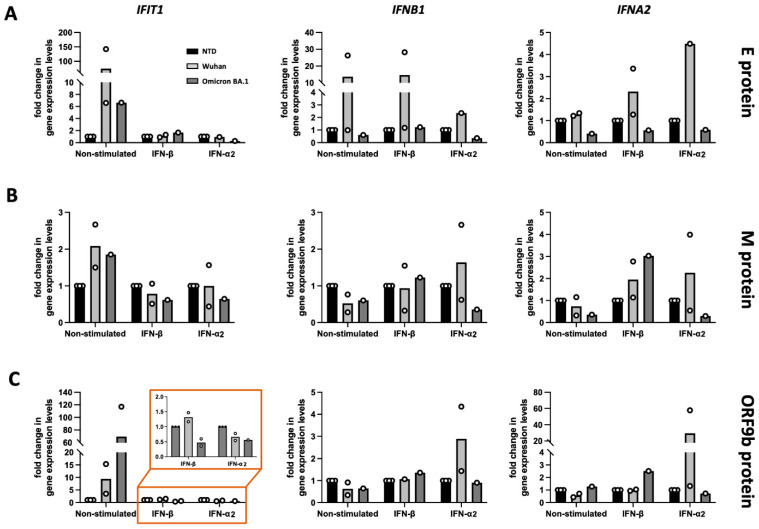
The effect of SARS-CoV-2 Envelope, Membrane, and ORF9b proteins from Wuhan and Omicron BA.1 variants on IFN-I response in HUVEC cells, with or without IFN stimulation. HUVEC cells were transduced with lentiviruses encoding the Envelope (**A**), Membrane (**B**), or ORF9b (**C**) proteins from the SARS-CoV-2 Wuhan (light grey bars) or Omicron BA.1 variants (dark grey bars). Non-transduced (NTD) cells were used as controls (black bars). Cells were either non-stimulated or stimulated with IFN-β or IFN-α2 for 24 h. Expression of IFIT1 (**left column**), IFNB1 (**middle column**), and IFNA2 (**right column**) was measured by qPCR. Fold-change in gene expression levels was calculated relative to non-transduced, non-stimulated cells. Dots indicate values obtained from independent experiments; bars show average over biological replicates. The data were obtained from six independent biological donors.

Even though considerable variability was observed between biological replicates in different donors, analysis of ISGs expression levels in transduced HUVEC cells revealed distinct patterns of ISG induction under non-stimulated and interferon-stimulated conditions (Figure 5).

Compared to the Wuhan strain, the Omicron variant of the Envelope protein (Figure 5A) exhibited diminished ability to induce ISG expression (IFIT1 and IFNB1) independently, as seen in the non-stimulated condition. Upon external stimulation with IFN-β or IFN-α2, the Omicron BA.1 variant did not amplify the interferon-induced signaling pathway, as evidenced by the attenuated transcription of IFIT1, IFNB1, and IFNA2, compared to the Wuhan variant of the Envelope protein. This suggests that the Envelope protein, particularly from the Omicron BA.1 variant, has evolved to avoid boosting interferon responses, which may reflect viral adaptation to evade host immune responses.

For the Membrane protein (Figure 5B), the differences between transduction and stimulations were minimal, except for a consistent reduction of IFIT1, IFNB1, and IFNA2 expression in IFN-α2 stimulated HUVECs expressing the Omicron variant. These findings suggest that the Membrane protein only plays a minor or context-dependent role in the modulation of interferon responses.

The Omicron ORF9b protein (Figure 5C) also displayed this phenotype; however, both Omicron and Wuhan variants induced IFIT1 expression in unstimulated but not in IFN-β or IFN-α2 stimulated HUVECs.

## 4. Discussion

The immune evasion strategies employed by SARS-CoV-2 remain one of the defining feature of viral fitness. Our study provides a comprehensive review and characterization of viral proteins that contribute to this process, pointing to both conserved and variant-specific immune modulation across the Wuhan and Omicron variants. Through a systematic literature review and experimental validation using luciferase-based assays and an endothelial cell model, we identified viral proteins that modulate IFN responses.

Our global screening approach using a dual-luciferase reporter assay quantitatively confirmed the well-characterized IFN antagonistic functions of several viral proteins while also revealing novel differences in strain-specific immune suppression. Consistent with previous reports on the Wuhan variant [44,80,107,110,169,170], Nsp1, Nsp5, Nsp14, and ORF6 emerged as potent suppressors of ISRE-driven IFN responses in both the Wuhan and Omicron variants. This supports their well-documented roles in shutting down host protein synthesis, blocking IFN-stimulated gene (ISG) expression, and preventing nuclear translocation of key transcription factors such as IRF3. Interestingly, we found that several viral proteins displayed different immune-suppressive capabilities between the two strains (Figure 3 and Figure 5), highlighting the adaptive evolution of SARS-CoV-2 in response to host immunity.

Among these, Nsp6 exhibited enhanced IFN suppression in the Omicron variant compared to the Wuhan strain (Figure 3A–D). This suggests that selective pressure on non-structural proteins has contributed to Omicron’s immune evasion capacity, beyond the extensive mutations observed in the Spike protein. In this cell line model, the Envelope protein plays a role in stringent IFN suppression in the Wuhan strain, which is pretty much conserved in Omicron. Given that the Envelope protein is implicated in viral assembly and release [52], as well as aiding in immune tolerance [53,54], conserved immune suppressive activity points to a selective pressure. Similarly, this pressure is also observed for the Membrane protein. In contrast, the accessory proteins ORF7a and ORF8 strongly inhibited IFN responses, and their sequences are highly conserved across the variants, suggesting a crucial role in interacting with host immune regulators and downregulating MHC-I or blocking STAT1 phosphorylation. These findings also raise an important question about whether Omicron’s reduced pathogenicity results directly from cumulative virus–host interactions contributing to an enhanced immune evasion phenotype.

The results from our cytotoxicity assay further emphasized the complexity of SARS-CoV-2-host interactions. While early expression of most viral proteins had minimal impact on cell viability at 8 h post-transfection, a subset of proteins (Nsp3, Nsp5, Nsp6, Nsp13, Nsp14, and ORF3a) exhibited significant cytotoxicity at 24 h. This delayed effect suggests that these proteins have a moderate effect and may disrupt host cell homeostasis over time, potentially contributing to the systemic inflammation and endothelial dysfunction observed in severe COVID-19 cases.

A key feature of SARS-CoV-2 pathogenesis is its disruption of endothelial function, which leads to thrombotic complications [11]. As a respiratory virus, besides infecting ACE2-positive cells, SARS-CoV-2 appears uniquely capable of infecting human ACE2-low endothelial cells through αV/β3 integrin-mediated endocytosis [171]. This invalidates a previous hypothesis that the virus does not effectively infect the blood vessel lining due to the relatively lower ACE2 receptor density on endothelial cell surfaces, as opposed to respiratory epithelial cells [172].

Additionally, our endothelial cell model allowed us to assess the direct impact of select viral proteins (Envelope, Membrane, ORF9b) on IFN responses in primary human endothelial cells. While leukocytes—especially mononuclear phagocytes (MPs), including monocytes, macrophages, and dendritic cells (DCs), as well as pDCs—are the primary producers of type I interferons (IFNs) in response to SARS-CoV-2 infection [173,174,175], endothelial cells, while not traditionally considered major IFN-I producers, play a crucial role in COVID-19 by acting as immune sentinels within the vascular system since they express PRRs [176,177]. They contribute to localized immune responses by producing low levels of IFNs that help regulate inflammation and maintain vascular integrity. SARS-CoV-2-mediated IFN suppression in endothelial cells impairs their ability to regulate inflammation, leading to increased vascular permeability and immune cell recruitment [178]. While leukocytes drive systemic IFN responses, endothelial cells orchestrate localized immune regulation, highlighting their distinct yet complementary roles in COVID-19 immunopathology.

Our findings show that the Envelope protein from the Omicron variant exhibits an inhibitory effect on IFN responses compared to the Wuhan strain. In contrast, ORF9b displayed a paradoxical phenotype—inducing IFIT1 expression in unstimulated conditions while suppressing it upon IFN stimulation. This suggests that ORF9b may function as an immune modulator rather than a strict inhibitor, dynamically adjusting host responses to favor viral persistence. While it blocks TBK1 phosphorylation by targeting TOM70 at the mitochondria [179,180,181] and binds NEMO (IKKγ), thereby inhibiting NF-kB activation [151], it seems that ORF9b plays an important role in endothelial dysfunction. ORF9b is reported to upregulate fibrinogen α, β, and γ—key prothrombotic factors that correlate with coagulation disorders and vascular inflammation in severe COVID-19 cases [155]. Fibrinogen is highly regulated by cytokines of the IL-6 family, which have been shown to be dramatically increased in patients with severe COVID-19 [182]. The elevated IL-6 levels in these patients likely contribute to the hypercoagulable state, exacerbating endothelial dysfunction and thrombosis, as high IL-6 are associated with higher cardiovascular disease risk [183]. ORF9b is also reported to impair cardiomyocytes metabolism [153] similar to the Membrane protein [50], which disrupts calcium handling and compromises cardiac contractility. The inclusion of the Membrane protein in our endothelial cell studies yielded less conclusive results, as both strains showed minimal differences in ISG induction. This suggests that while the Membrane protein may contribute to immune evasion, its role in endothelial dysfunction likely occurs through alternative pathways.

While endothelial dysfunction is a shared mechanism between SARS-CoV-2 variants, the Wuhan strain was more strongly associated with severe thrombotic events than Omicron. Studies have shown that earlier variants, including the Wuhan strain, exhibited a more pronounced hypercoagulable state with increased clot formation potential compared to Omicron [184,185]. However, endothelial involvement in immune modulation remains relevant for both variants, as our study focuses on IFN suppression rather than direct thrombotic outcomes.

While the study presents a comprehensive comparative analysis of SARS-CoV-2 immune modulation across the Wuhan and Omicron variants and is indicative of the need to study their impact on the vascular system, there are several limitations. First, the use of overexpression systems in transduced endothelial cells, while informative, may not fully replicate the physiological context of viral infection, where protein expression levels and timing are tightly regulated. Second, ISRE-driven luciferase, although valuable, provides only a snapshot of IFN pathway activation and does not capture the broader landscape of immune signaling dysregulation or downstream cytokine responses. Previous studies in cell lines, which tested only viral proteins from the Wuhan variant, expanded their scope by examining different steps of the immune signaling pathways, including RIG-I, MDA5, MAVS, TRIF, IRF3, or STAT1 and STAT2 [30,44,80,110,169]. Additionally, our study lacks further mechanistic validation of the effect on ISGs’ transcription in primary endothelial cells, which are physiologically relevant to SARS-CoV-2 infection and pathogenesis. However, experiments involving primary endothelial cells, such as human umbilical endothelial cells (HUVECs), are technically challenging and time-consuming, presenting an additional experimental barrier. The absence of in vivo models or patient-derived samples further restricts the translational relevance of the findings. Finally, while the evolutionary divergence of proteins such as the Envelope, Membrane, and Spike proteins is intriguing, the mechanism of their altered immune evasion capabilities remains insufficiently explored. Addressing these limitations in future studies would significantly enhance the robustness and applicability of the findings.

Another limitation concerns the expression of membrane-bound proteins. The S, M, and E proteins naturally undergo glycosylation and intracellular trafficking, which may not be fully replicated in our recombinant expression system. While we used Strep-tagged constructs to detect proper expression, the potential lack of native post-translational modifications could affect their interaction with cellular immune pathways. Future work using live-virus models would help refine our understanding of these processes.

Nevertheless, these findings primarily aim to contribute a baseline of understanding in the SARS-CoV-2 evolution and their implications in vascular pathologies. It remains unclear whether the changes between variants reflect a trade-off between immune evasion and transmissibility, or whether additional factors such as host immune memory and vaccination have influenced the functional evolution of SARS-CoV-2 proteins. This study provides two key contributions: (1) a comprehensive assessment of SARS-CoV-2 proteins and their immune-modulatory effects across two major variants; (2) an underexplored avenue of SARS-CoV-2 endothelial dysfunction research that warrants further investigation.

## Data Availability

Dataset available on request from the authors.

## Supplementary Materials

The following supporting information can be downloaded at: https://www.mdpi.com/article/10.3390/v17040569/s1, Figure S1: Lentiviral Transduction of Primary Human Umbilical Vein Endothelial Cells (HUVEC) and Interferon-Stimulated Gene Expression Analysis; Table S1: SARS-CoV-2 viral proteins and their mechanism of action.
