# Supplementary material for: Impact of SARS-CoV-2 Wuhan and Omicron Variant Proteins on Type I Interferon Response
Source: Viruses. 2025 Apr 15;17(4):569. doi: 10.3390/v17040569 (PMC12031613; doi:10.3390/v17040569)
Supplement: Supplementary file 1 [file viruses-17-00569-s001.zip › viruses-3546562-supplementary.pdf]

Supplemental material

Table S1 SARS-CoV-2 viral proteins and their mechanism of action

Gene	Viral protein	Role
ORF1a	Nsp1	**Host translation inhibitor**<br>Shuts down the host protein synthesis machinery by interacting with the 40S subunit of the ribosomes cleaving host RNA.
	Nsp2	**Proofreading replicase**
	Nsp3	**Papain like protease (PLpro).**<br>Cleaves viral polyprotein (processes pp1a and pp1ab).
	Nsp4	Forms complexes with Nsp3 and Nsp6 (double-membrane vesicle), required for viral replication. Helps tying RTC to ER.
	Nsp5	**Chymotrypsin-like protease (3CLpro) or Main protease (Mpro)**<br>Processes viral polyproteins into functional units.
	Nsp6	Complexes with Nsp3 and Nsp4 (double-membrane vesicle), required for viral replication.
	Nsp7	**The primase complex**<br>Forms the primase complexes with Nsp8 to act as cofactor for the RNA-dependent RNA polymerase (Nsp12) during RNA synthesis
	Nsp8	**The primase complex**<br>Forms the primase complexes with Nsp7 to act as cofactor for the RNA-dependent RNA polymerase (Nsp12) during RNA synthesis
	Nsp9	Exact role unclear
	Nsp10	**RNA capping machinery**<br>Triggers RNA capping machinery by acting as a co-factor to Nsp14 (exoribonuclease) and Nsp16 (2'-O-methyltransferase).
	Nsp11	Exact role unclear
ORF1b	Nsp12	**RNA-dependent RNA polymerase (RdRp)**<br>Catalyzes viral RNA synthesis
	Nsp13	**Helicase**<br>Part of RNA capping machinery. Unwinds viral RNA during replication. Exhibits NTPase activity.
	Nsp14	**Proofreading exoribonuclease (ExoN)**<br>Ensures fidelity of RNA during synthesis via proofreading and has a role in capping through its function as a guanine-N7 methyltransferase
	Nsp15	**Uridylate-specific endoribonuclease (EndU)**<br>Cleaves viral RNA intermediates.
	Nsp16	**2'-O-ribose methyl transferase**<br>Part of RNA capping machinery.
ORF2	S protein	**Spike protein**<br>Mediates viral entry by binding to ACE2 on host cells. Facilitates membrane fusion via cleavage by host proteases like TMPRSS2 and furin.
ORF3a	ORF3a	**Virioporins**<br>Forms ion channels (virioporins) to allow safe import of virions via lysosomes by deacidifying the lysosome.
ORF4	E protein	**Envelope protein**<br>Essential for viral assembly and budding. Forms viroporins and acts as a ion channel (virioporin) to maintain membrane potential.
ORF5	M protein	**Membrane**<br>Plays a central role in virion assembly by interacting with other structural proteins. Shapes the viral envelope.
ORF6	ORF6	Role in immune modulation
ORF7a	ORF7a	Inhibits BST-2 (tetherin) glycosylation and helping virion egress.
ORF7b	ORF7b	May play a role in enhancing virion stability
ORF8	ORF8	Role in immune modulation
ORF9	N protein	**Nucleocapsid**<br>Stabilizes viral RNA, by encapsulating the viral RNA to form the ribonucleoprotein complex. Enhances viral replication and transcription.
	ORF9b	Role in immune modulation
ORF10	ORF10	**Ubiquitin ligase**<br>Unknown mechanism

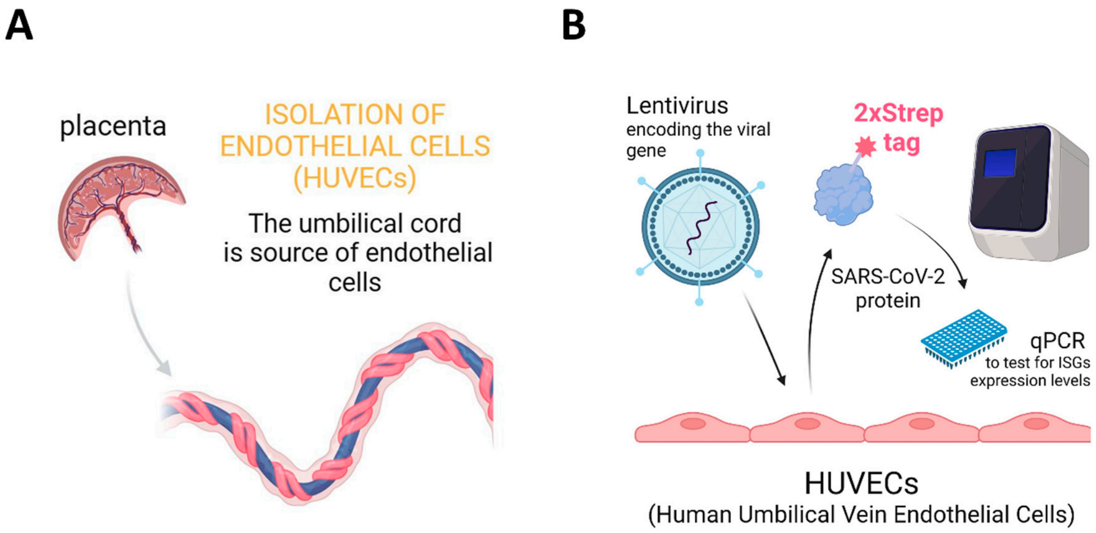

Figure S1 Lentiviral Transduction of Primary Human Umbilical Vein Endothelial Cells (HUVEC) and Interferon-Stimulated Gene Expression Analysis *(A) HUVEC were freshly isolated from donated human umbilical cords, expanded on fibronectin-coated plates, and maintained in endothelial cell culture medium. (B) Lentiviruses encoding Strep II-tagged SARS-CoV-2 proteins (Wuhan and Omicron variants) were produced in HEK293T cells and titrated. HUVEC were transduced in fibronectin-coated plates using polybrene, then stimulated with IFN-α2 (500 IU/mL) or IFN-β (1000 IU/mL) 24 hours post-transduction. Non-stimulated and non-transduced controls were included. Cells were lysed and total RNA was extracted for qPCR analysis of interferon-stimulated genes (IFNβ, IFNα, IFIT1) to assess the impact of SARS-CoV-2 proteins on the IFN response.*
